# Prediction of exertional lower extremity musculoskeletal injury in tactical populations: protocol for a systematic review and planned meta-analysis of prospective studies from 1955 to 2018

**DOI:** 10.1186/s13643-018-0883-6

**Published:** 2018-12-23

**Authors:** Shawn D. Flanagan, Kellen T. Krajewski, Aaron M. Sinnott, Caleb D. Johnson, Shawn R. Eagle, Alice D. LaGoy, Meaghan E. Beckner, Anne Z. Beethe, Rose Turner, Mita T. Lovalekar, Courtenay Dunn-Lewis, Chris Connaboy, Bradley C. Nindl

**Affiliations:** 1 Neuromuscular Research Laboratory and Warrior Human Performance Research Center, Department of Sports Medicine and Nutrition, School of Health and Rehabilitation Sciences, University of Pittsburgh, Pittsburgh, PA USA; 2 Health Sciences Library System, University of Pittsburgh, Pittsburgh, PA USA; 3 Department of Health and Physical Activity, School of Education, University of Pittsburgh, Pittsburgh, PA USA

**Keywords:** Biomechanics, Epidemiology, Exercise, Prediction, Knee, Overuse, Performance, Rehabilitation, Risk factors, Training

## Abstract

**Background:**

Musculoskeletal injuries (MSI) represent more than half of all injuries in tactical populations (i.e., military service and public safety workers including police, firefighters, emergency medical services (EMS)). Most lower extremity MSIs result from physical exertion during training, occupational tasks, and recreation. Such exertional lower extremity injuries (ELEI) produce a significant human and financial cost. Accordingly, significant efforts have been made to identify sensitive, specific, and reliable predictors of ELEI. There is a need to synthesize and evaluate the predictive value of risk factors for ELEI while addressing the influence of occupation, sex, exposure, injury characteristics, and study quality. Therefore, the purpose of this systematic review and planned meta-analysis is to evaluate risk factors for ELEI in tactical populations.

**Methods:**

After the development of a search strategy, comprehensive searches will be conducted in MEDLINE, EMBASE, Cochrane, and CINAHL databases. Articles will be screened with a multi-user process and delimited to prospective comparative cohort studies that directly measure injury occurrence in the target population(s). Extracted data will be synthesized and assessed for reporting bias, meta-bias, and overall quality, with subgroup analyses to determine the influence of participant, injury, and exposure characteristics in addition to study quality.

**Discussion:**

This systematic review and planned meta-analysis will comprehensively evaluate ELEI risk factors. Information gained will inform injury prevention protocols, facilitate the use of improved measurements, and identify requirements for future research.

**Trial Registration:**

The systematic review protocol was registered with the International Prospective Register of Systematic Reviews (PROSPERO) on 3 Jan 2018 (registration number CRD42018056977).

**Electronic supplementary material:**

The online version of this article (10.1186/s13643-018-0883-6) contains supplementary material, which is available to authorized users.

## Background

The threat posed by MSI to the readiness, performance, and long-term health of tactical populations is well-recognized [1–4]. In the U.S. military alone, musculoskeletal injury (MSI) is responsible for nearly 75% of limited duty cases and costs billions of dollars annually [5–9]. In public safety workers, MSI accounts for more than half of all injuries [2, 10, 11] with a 4- to 40-fold increase in risk compared to other occupations [4, 12]. The long-term implications of MSI were recently highlighted in a systematic review that identified military, police, and firefighters as being several times more likely to experience knee and hip osteoarthritis compared to controls [13]. Given the prevalence and burden of MSI in tactical populations, significant attention has been placed on the identification of risk factors that predict injury [3–5, 14].

Physical exertion (e.g., training, occupational tasks, and recreation) is essential for the development and maintenance of physical performance and fitness, core occupational competencies for tactical populations [8, 15, 16]. Nevertheless, physical exertion is also the leading cause of MSI [6, 7, 14, 17, 18] with the lower extremities most frequently affected [4, 5, 19–22]. Such exertional lower body injury (ELEI) is associated with previous injury [23–30], female sex [34–43], various lifestyle factors (e.g., smoking) [43], genetic variation in musculoskeletal structures [44–48], neurocognitive function [49–54], and lower physical fitness [30–33]. Paradoxically, while physical fitness is inversely related to ELEI risk, higher training volumes used to improve physical fitness also increase injury risk [55, 56]. These observations underscore the need to synthesize and evaluate specific aspects of physical exertion (e.g., training mode, volume, load) alone and in combination with other individual, behavioral, environmental, or occupational factors that may moderate ELEI risk in tactical populations [3, 25, 30, 57–63].

A 2003 review of risk factors for lower extremity injury identified a number of limitations in the literature base, including (1) inadequate statistical power, (2) need for comparisons based on sex, (3) confound of unadjusted exposure levels, and (4) inconsistent injury classification schemes [64]. Similarly, the development of increasingly advanced (and accessible) technologies has provided new ways to predict and diagnose MSI [44, 45, 65, 66]. As a result, myriad techniques and measures have been promoted as superior predictors of ELEI with little validation and considerable risk of misuse. Thus, there is a need to quantitatively synthesize new evidence, while addressing known limitations of the literature base [13, 67].

In this systematic review and planned meta-analysis, we evaluate prospective studies to determine the predictive value of risk factors for ELEI in tactical populations. A secondary purpose is to perform subgroup analyses to evaluate risk factors based on participant, exposure, injury, and study characteristics. The synthesis of rigorous evidence will clarify generalized predictors of ELEI and identify factors specific to different tactical populations. The findings of this review will support efforts to identify targets for ELEI prevention and the optimization of physical training.

### Objectives

The primary objective of this systematic review is to evaluate predictors of ELEI in military and public safety populations. The proposed systematic review will address the following topics:

1. Determine the most sensitive and specific risk factors for ELEI in military and public safety populations.
2. Assess the influence of different participant and study characteristics on the prediction of ELEI and statistical heterogeneity.

## Methods

### Eligibility criteria

Studies will be selected based on the following criteria for study design, participants, interventions, comparators, outcomes, and other study traits.

#### Study designs

To identify, synthesize, and evaluate risk factors for ELEI, prospective comparative cohort studies will be included. Prospective observational studies will include direct between-group comparisons; single-arm studies will be excluded (e.g., benchmarking, simulated comparisons). Randomized controlled trials (RCTs), retrospective studies, cross-sectional studies, case-control, nested case-control, case reports and series, ideas, opinions, editorials, animal research, and in vitro research will be excluded.

#### Participants

Studies on adults aged 18 years or older will be included. Participants must be active military service members or public safety workers (including police, firefighters, and emergency medical services (EMS)). International military members that meet the national age requirement will be included. Studies of the target populations after retirement will be excluded.

#### Interventions

Risk factors for ELEI in tactical populations will be evaluated. Acceptable risk factors include continuous, categorical, objective, subjective, modifiable, non-modifiable, internal, or external variables associated with modified risk of injury. Injuries will be identified in accordance with terminology provided by the International Classification of Diseases (ICD-9-M and ICD 10) [68, 69] and National Institute for Occupational Safety 111 and Health (NIOSH) [1]. Qualifying injuries will include fractures, derangements, dislocations, sprains, strains, and other injuries or disorders of the muscles, nerves, tendons, joints, or cartilage of the lower limbs. Such injuries must be caused, precipitated, or exacerbated by sudden or prolonged exertion involving movement repetition, force, vibration, or adverse biomechanics. Injuries resulting from falls (parachuting), motor vehicle accidents, violence, external contact, and war will be excluded.

#### Comparators

For the analysis of prospective observational studies, controls will include participants who do not experience ELEI.

#### Outcomes

Outcomes and their definitions will be collected as reported in individual studies. Dichotomous data will be extracted, reflecting the nature of the disease in question (ELEI incidence). Anticipated measures include incidence, odds ratios, risk ratios, and likelihood ratios. Outcomes may be derived from self-report, medical records, behavioral performance, medical imaging, physiological measurements, and biological specimens. Studies that do not report injury incidence will be excluded. Outcome measurements specific to ELEI will be extracted. Composite and upper body MSI will not be included, and studies that do not report or provide lower body-specific data will be excluded. If ELEI is subdivided by structure, tissue, category, or other factors, all levels of data will be extracted and pooled depending on injury classification.

#### Timing

Studies with a minimum surveillance period of 3 months will be prioritized. A 3-month surveillance period was selected to ensure the detection of ELEIs such as tibial stress fractures or subsequent injuries that develop over prolonged periods of time and thus likely underreported during shorter observation periods [68, 69]. Studies with surveillance durations of at least 2 months will be considered if otherwise acceptable and of sufficient quality as determined by the review team.

#### Setting

Study setting is unrestricted.

#### Language

Articles reported in English will be included. When applicable, studies involving predominantly non-English speaking populations will be included.

#### Other review eligibility criteria

Study inclusion will not be restricted by geographic region. Inclusion will be limited to original research articles published in peer-reviewed journals. Abstracts, unpublished data, commentaries, letters, and conference proceedings will be screened. Data from 1955 to 2018 will be included and evaluated for systematic variation of effect estimates associated with publication age. If affected by publication age, effect estimates may be normalized or subgrouped and analyzed separately.

### Information sources

MEDLINE, EMBASE, Cochrane, and CINAHL electronic databases will be searched. We will also search the reference lists of included publications, relevant reviews, and gray literature. Article listings in the top five journals of included studies will be searched from 2003 to 2018. The final reference list will be circulated among the review team and external experts identified by the team. The search strategy will meet IOM Standards for Systematic Reviews [70].

### Search strategy

The search strategy will use medical subject headings (MeSH) and text words that identify the target populations, injuries and structures, exposures and practices, and analysis features relevant to injury prediction. Searches will be limited to original human research articles published in peer-reviewed journals in English. Searches will be limited to 1955–2018, but not limited for study design. The MEDLINE search strategy will be developed by a health sciences librarian with expertise in systematic review searches (RT) in collaboration with the project team and reviewed by an additional Librarian (see Additional file 1 for search strategy). The MEDLINE search strategy will be adapted for use with EMBASE, Cochrane, and CINAHL databases. The electronic database search will be updated and re-run before the final analysis to capture new studies and confirm retrieval of a high proportion of eligible studies. PROSPERO will be searched for ongoing and completed reviews at the beginning and end of the review process. Recently completed systematic reviews will be searched for new articles.

### Study records

#### Data management

Citation abstracts and full-text articles captured during the literature search will be uploaded into Distiller Systematic Review (DSR) software for screening. Before DSR upload, duplicate search results will be removed using Endnote reference management software. When multiple reports on a study are identified, the study team will assess the consistency of reported study design, sample size, outcome (s), and statistical test (s). All relevant original information will be extracted and collated. In the event of multiple versions, the initial version will be retained. The inclusion and exclusion criteria will be developed into a series of forms for article screening (see Additional file 2 for screening forms). The forms will be tested and refined before formal screening. New members of the team will be trained on the content area and DSR software prior to participation.

#### Selection process

Titles of studies retrieved from the search will be independently screened by two reviewers for relevance to the prediction of ELEI. Reviewers will be blind to study author (s), journal title, and institution. For level two screening, abstracts will be reviewed for requisite study outcomes. Level three screening will confirm the eligibility of the participant population. After the first three levels of screening, if the initial inclusion criteria are met or inconclusive, the full article will be obtained. At the fourth screening level, the full inclusion criteria will be applied to the text. When questions about eligibility persist, study authors will be contacted for additional information. If the study author does not respond to contact for additional information or the response is deemed inadequate, the study will be screened and reason (s) noted. Disagreements about inclusion will be resolved through discussion or final review by a third author. Reason (s) for excluding articles will be reported. Inter-rater agreement will be analyzed after the completion of three selection process procedures: (1) initial assessment test, (2) refinement, and (3) formal screening process.

#### Data collection process

Data will be extracted in duplicate by two independent reviewers using standardized forms in DSR. To improve consistency, data extraction forms will be pre-piloted in conjunction with the selection forms (see Additional file 3 for data extraction forms). Disagreements will be resolved by a third reviewer (SF, CC, CD, and ML). When data from primary studies do not present all necessary data, the study authors will be contacted and asked to provide the missing data. We will attempt to contact primary authors up to three times by email or phone. In the event of uncertainty or missing data, failure to respond, comply, or follow-up will result in study exclusion. The original study authors will also be contacted to confirm the accuracy of the final extracted data. When multiple reports of a single study are identified, non-duplicated information will be collated if the observation periods are the same. Otherwise, data from the initial report will be included, with the exclusion of duplicates noted.

### Data items

Extracted data will include study characteristics (study setting and design, sample size, duration of follow-up, methods and intervention details, measurement techniques, and time points), participant and exposure characteristics (occupation, duration of participation, demographic, anthropometric, biological, workload, history, fitness, exertional characteristics), injury characteristics (definition, reporting method, characteristics, location, and subtype, indicators of acceptability), author interpretation (s) of findings, suggested mechanisms of action, potential confounders, and information for the assessment of risk of bias.

Outcomes specific to anatomic structures (e.g., tibia, Achilles tendon, soleus muscle) and disorders (e.g., stress fracture, tendinopathy, and muscle strain) will be collected for further analysis or aggregation. In the health and rehabilitation science literature, MSI is often classified as non-traumatic (overuse) or traumatic (acute) and risk factors are commonly classified as extrinsic/intrinsic and modifiable/non-modifiable. If injury descriptions are consistent across studies, injuries will be subtyped accordingly. Measures of central tendency and variability will be extracted from figures when necessary using plot digitization software. If effect sizes cannot be calculated, study authors will be contacted for additional information.

### Outcomes and prioritization

#### Primary outcome

The primary outcome will be relative measures of effect (i.e., risk ratios, odds ratios, hazard ratios, etc.) based on ELEI incidence rates. All outcomes will be calculated from the total number of participants at each measurement time point. The use of a dichotomous outcome enhances comprehension and is therefore preferred.

### Risk of bias

Two blinded reviewers will independently assess the risk of bias for each included study. Disagreement between the reviewers will be resolved after discussion or final decision by a third reviewer (SF, CC, CD, and ML). To assess the risk of study bias and overall study quality, the following factors will be considered: study participation, study attrition, prognostic factor measurement, outcome measurement, potential confounding factors, statistical analyses, and reporting. Descriptions provided in the study will be collected and scored using the Quality in Prognostic Studies (QUIPS) instrument [71]. The risk of bias will be summarized graphically across studies. The synthesis will include a subgroup analysis to examine the influence of study quality on the overall findings of the review. Low-quality studies may be excluded from the final analysis.

### Data synthesis

Under the assumption of sufficient homogeneity, quantitative analysis of aggregate participant data will be conducted. The planned analytical approach will include a narrative synthesis of the findings from the included studies, structured around the primary predictors of injury, injury characteristics, and target population characteristics. Study results will be dichotomized and expressed as risk ratios (RR) with 95% confidence intervals (CI). Original study authors will be contacted when there are missing data.

The results of included studies will be pooled and analyzed using stratified random-effects meta-analysis in specialized meta-analysis software (RevMAN v5.3; The Cochrane Collaboration). Subgroup and meta-regression (Stata, StataCorp) analyses will be used to determine whether prediction estimates are influenced by potential effect modifiers such as study characteristics and participants characteristics (see the “Subgroup analysis” section below). Statistical heterogeneity will be determined with chi-square (*χ*^2^) and *I*^2^ (0–100%) statistics, with *χ*^2^ *p* > 0.10 and *I*^2^ > 50% indicative of substantial heterogeneity. Between-study variance (_*T*_^2^) will be assessed in relation to effect size estimates. In the event of substantial or high heterogeneity (*I*^2^ > 75%) [72], sensitivity analysis will be conducted as indicated by subgroup comparisons and QUIPS checklists.

Review results will be ordered by objective. Important comparisons will also be presented based on the availability of data. Results will be presented in narrative text, tables, and figures. Depending on the outcome of sensitivity and subgroup analyses, studies with a high risk of bias may be omitted from the final meta-analysis.

#### Subgroup analysis

If the necessary data are available, sources of between-study heterogeneity and the robustness of the meta-analysis will be investigated with sensitivity and subgroup analyses. Potential risk factors and comparisons will be considered on a case-by-case basis depending on the literature base with the maximal feasible amount of data extracted for the following (likely) subgroup analyses:

1. Participant characteristics (sex, age, occupation)
2. Injury location, structure, class (e.g., ankle, calcaneofibular ligament, sprain)
3. Injury subtype (acute vs. non-traumatic)
4. Risk factor presence, magnitude, or class (extrinsic, intrinsic, modifiable, non-modifiable)
5. Surveillance period
6. Study-specific factors
  - Sample size
  - Overall study quality
  - Risk of bias (e.g., all studies versus low bias studies only)
  - Publication age

### Meta-bias

The possibility of publication and outcome reporting bias will be explored with funnel plots and the QUIPS checklist. Small sample bias will be assessed by comparing fixed effect estimates with the random effects model. The results may be used to re-weigh studies or conduct further subgroup analysis. The analysis and reporting conducted in this review will comply with guidelines provided by the Measurement Tool to Assess Systematic Reviews (AMSTAR-2) [73].

### Confidence in cumulative estimate

The overall quality of the body of evidence will be determined with the Grading of Recommendations Assessment, Development, and Evaluation (GRADE) approach [74]. Studies excluded from the meta-analysis will be excluded from the GRADE assessment. GRADE results will be used to inform conclusions on the overall strength of predictors of ELEI in tactical populations.

## Discussion

This systematic review and planned meta-analysis will evaluate the independent and collective predictive strength of risk factors for ELEI in tactical populations, including Military Service Members, Firefighters, Police, and EMS. Study inclusion will be determined through a rigorous multi-level screening process with comprehensive assessments for quality and bias. Valid and reliable predictors of ELEI will be identified in high-quality prospective studies with direct measures of injury occurrence. Subgroup analyses will examine the influence of prominent injury characteristics, risk factors, and potential sources of heterogeneity. The analysis will provide the fields of health and rehabilitation sciences and human performance optimization with a robust and comprehensive resource to inform future practices and research initiatives. The authors of this effort are committed to a rigorous and transparent systematic review process. For information on compliance with PRIMSA-P review guidelines, see Additional file 4.

## Additional files

Additional file 1: MEDLINE search strategy (PDF) – terms and connectors for literature search. (PDF 25 kb)

Additional file 2: Screening form (PDF) – questions and notes for each level of screening. (PDF 136 kb)

Additional file 3: Data extraction form (PDF) – topics for data extraction, synthesis, and quality assessment. (PDF 18 kb)

Additional file 4: PRISMA-P form (PDF) – populated PRISMA-P checklist systematic review protocol. (PDF 124 kb)

## Abbreviations

AMSTAR-2: Measurement Tool to Assess Systematic Reviews
CI: Confidence intervals
CINAHL: Cumulative Index to Nursing and Allied Health Literature
DSR: Distiller Systematic Review
ELEI: Exertional lower body injury
EMBASE: Excerpta Medica database
EMS: Emergency medical service
GRADE: Grading of Recommendations Assessment, Development, and Evaluation
ICD: International Classification of Diseases
IOM: Institute of Medicine
MEDLINE: U.S. National Library of Medicine bibliographic database
MeSH: Medical subject headings
MSI: Musculoskeletal injury
NIOSH: National Institute for Occupational Safety and Health
PRISMA: Preferred Reporting Items for Systematic Reviews and Meta-Analyses
PROSPERO: International prospective register of systematic reviews
QUIPS: Quality in Prognostic Studies
RCT: Randomized controlled trial
RR: Risk ratio
