# Supplementary material for: Prediction of exertional lower extremity musculoskeletal injury in tactical populations: protocol for a systematic review and planned meta-analysis of prospective studies from 1955 to 2018
Source: Syst Rev. 2018 Dec 23;7:244. doi: 10.1186/s13643-018-0883-6 (PMC6304230; doi:10.1186/s13643-018-0883-6)
Supplement: Supplementary file 3 — Data extraction form (PDF) – topics for data extraction, synthesis, and quality assessment. (PDF 18 kb) [file 13643_2018_883_MOESM3_ESM.pdf]

ADDITIONAL FILE 3: Data Extraction Items

1. Study Characteristics

a. Setting
b. Study Design
c. Sample Size
d. Duration of follow-up/surveillance period
e. Type of control
f. Methods and intervention details
g. Measurement techniques
h. Measurement time points

2. Potential risk factors

a. Occupation
b. Duration of service/participation/deployments
c. Demographic, Anthropometric, and Biological
    i. Sex
    ii. Age
    iii. Race
    iv. Height
    v. Somatotype
    vi. Body composition (fat mass, lean mass, fat percentage, body mass index)
    vii. Bone composition (density, mass)
d. Workload
    i. Running mileage
    ii. Dosage and timing of activity exposure
    iii. Load carriage magnitude and frequency
    iv. Footwear
e. Personal History
    i. Physical activity
    ii. Previous injury
    iii. Smoking
    iv. Medication use
    v. Vitamin D physiology
    vi. Sleep quality/quantity
    vii. Mental/behavioral health
f. Physical Fitness
    i. Aerobic capacity
    ii. Anaerobic capacity

iii. Flexibility
iv. Maximal voluntary dynamic or isometric muscular strength
v. Maximal muscular power
vi. Movement Screen (e.g. FMS)
vii. Muscular Endurance

3. Risk factor class
  a. Extrinsic modifiable, extrinsic non-modifiable, intrinsic modifiable, intrinsic non-modifiable

4. Outcomes
  a. Injury characteristics
    i. Definition
    ii. Structure(s) or location(s)
    iii. Disorder(s)
    iv. Classification (e.g. overuse vs. acute)

5. Primary study conclusions

6. Suggested mechanisms to explain relationship risk factors and injury

7. Potential confounds

8. Items to complete the following checklists:
  a. Quality in Prognostic Studies (QUIPS) instrument
  b. Grading of Recommendations Assessment, Development, and Evaluation (GRADE)
